# Molecular Characterization of *Staphylococcus aureus* Isolated from Human and Food Samples in Northern Algeria

**DOI:** 10.3390/pathogens10101276

**Published:** 2021-10-03

**Authors:** Rachid Achek, Hosny El-Adawy, Helmut Hotzel, Ashraf Hendam, Herbert Tomaso, Ralf Ehricht, Heinrich Neubauer, Ibrahim Nabi, Taha Mossadak Hamdi, Stefan Monecke

**Affiliations:** 1 Faculty of Nature and Life and Earth Sciences, Djilali-Bounaama University, Soufay, Khemis-Miliana 44225, Algeria; achekrachid@gmail.com; 2 Laboratory of Food Hygiene and Quality Assurance System, High National Veterinary School, Oued Smar, Algiers 16059, Algeria; moussahamdi@hotmail.com; 3 Institute of Bacterial Infections and Zoonoses, Friedrich-Loeffler-Institut, 07743 Jena, Germany; helmut.hotzel@fli.de (H.H.); Herbert.tomaso@fli.de (H.T.); Heinrich.neubauer@fli.de (H.N.); 4 Faculty of Veterinary Medicine, Kafrelsheikh University, Kafr El-Sheikh 35516, Egypt; 5 Climate Change Information Center, Renewable Energy and Expert Systems (CCICREES), Agricultural Research Center, 9 Algamaa Street, Giza 12619, Egypt; a_hendam@hotmail.com; 6 Leibniz Institute of Photonic Technology (IPHT), 07745 Jena, Germany; ralf.ehricht@leibniz-ipht.de (R.E.); stefan.monecke@leibniz-ipht.de (S.M.); 7 InfectoGnostics Research Campus Jena e. V., 07743 Jena, Germany; 8 Institute of Physical Chemistry, Friedrich Schiller University Jena, 07743 Jena, Germany; 9 Faculty of Sciences, Yahia Farès University, Urban Pole, Médéa 26000, Algeria; ibrahiim.nabi@gmail.com; 10 Institute for Medical Microbiology and Virology, Dresden University Hospital, 01307 Dresden, Germany

**Keywords:** *Staphylococcus*, microarray assay, virulence genes, antibiotic resistance, Algeria

## Abstract

*Staphylococcus aureus* is a commensal resident of the skin and nasal cavities of humans and can cause various infections. Some toxigenic strains can contaminate food matrices and cause foodborne intoxications. The present study aimed to provide relevant information (clonal complex lineages, *agr* types, virulence and antimicrobial resistance-associated genes) based on DNA microarray analyses as well as the origins and dissemination of several circulating clones of 60 *Staphylococcus aureus* isolated from food matrices (*n* = 24), clinical samples (*n* = 20), and nasal carriers (*n* = 16) in northern Algeria. *Staphylococcus aureus* were genotyped into 14 different clonal complexes. Out of 60 *S. aureus*, 13 and 10 isolates belonged to CC1-MSSA and CC97-MSSA, respectively. The CC 80-MRSA-IV was the predominant *S. aureus* strain in clinical isolates. The accessory gene regulator allele *agr* group III was mainly found among clinical isolates (70.4%). Panton–Valentine leukocidin genes *luk*F/*luk*S-PV were detected in 13.3% of isolates that all belonged to CC80-MRSA. The *luk*F*/*S-*hlg*, *hlg*A, and *hla* genes encoding for hemolysins and leucocidin components were detected in all *Staphylococcus* *aureus* isolates. Clinical and food isolates harbored more often the antibiotic resistance genes markers. Seventeen (28.3%) methicillin-resistant *Staphylococcus aureus* carrying the *mec*A gene localized on a SCC*mec* type IV element were identified. The penicillinase operon (*bla*Z/I/R) was found in 71.7% (43/60) of isolates. Food isolates belonging to CC97-MSSA carried several antibiotic resistance genes (*bla*Z, *erm*B, *aph*A3, *sat*, *tet*M, and *tet*K). The results of this study showed that all clones were found in their typical host, but interestingly, some nasal carriers had isolates assigned to CC705 thought to be absent in humans. The detection of MRSA strains among food isolates should be considered as a potential public health risk. Therefore, controlling the antibiotics prescription for a rational use in human and animal infections is mandatory.

## 1. Introduction

*Staphylococcus aureus* is a Gram-positive bacterium, considered as a commensal resident of the skin and nasal cavities of humans and animals. This bacterium can spread from its habitual niches to other parts of the body and cause various clinical infections [1,2] and foodborne intoxications [3].

The pathogenic potential of *S. aureus* is essentially related to an arsenal of virulence factors and the capacity of acquiring resistance to different antibiotics [4,5]. The emergence of resistance to methicillin, and beta-lactams in general, has made methicillin-resistant *S. aureus* (MRSA) a serious public health problem [6].

The genetic background of *S. aureus* was intensively investigated using different typing tools such as pulsed-field gel electrophoresis (PFGE), *spa*-typing, multilocus sequence typing (MLST), whole genome sequencing, and DNA microarray-based analysis [7,8,9]. The study of the clonal diversity among *S. aureus* using molecular techniques contributed to our understanding of the genetic diversity of *S. aureus* and provided insights into the origin and spread of MRSA into humans and animals [10,11].

The clonal complexes (CC) CC5, CC8, CC22, CC30, and CC45, are typically hospital-associated MRSA (HA-MRSA) infections [11], while the lineages CC1, CC8, CC30 and CC80 have found to be mainly community-acquired MRSA (CA-MRSA) infections [12]. CC398 and CC9 have emerged as livestock-associated MRSA (LA-MRSA) [13,14].

The spread of specific clones differed depending on geographical regions. The sequence type (ST) ST80 was predominant in North African countries [15,16,17]. ST 8 (USA300) and ST1 (USA400) are prevalent in North America [18] and the Panton–Valentine leukocidin (PVL) positive ST93, known as “Queensland CA-MRSA”, in Australia [19]. However, some clones or lineages of *S. aureus* are not restricted to a specific host and can be found to colonize or cause infections in a broad variety of animal species, including humans [20,21]. The CC398 is associated with livestock, but is also able to colonize humans [22].

Panton–Valentine leukocidin (PVL) is a phage-borne toxin killing immune cells and causing tissue necrosis. PVL is a bi-component toxin forming polymeric pores in the membranes of target cells. It is structurally related to *luk*M/*luk*F-P83 from ruminant strains of *S. aureus*, which is also phage-borne. Other, similar bicomponent leukocidins in *S. aureus* are l*uk*F/S-*hlg* (on the gamma hemolysin locus), *luk*D/E (on a genomic island), and *luk*A/B (synonyms: *luk*X/Y, l*uk*G/H), which belong to the core genomic and can be considered as a species-specific marker of *S. aureus*. PVL genes are frequently found among community-acquired MRSA strains [23]. These strains are typically associated with severe skin and soft tissue infections [24].

The emergence of PVL-positive MRSA was previously detected in hospitals and communities in northern Africa in Algeria, Tunisia, and Egypt [16,17,25], in central Africa in São Tome and Príncipe, Nigeria, Ghana, and Kenya [26,27,28,29]) and in southern African countries such as South Africa [30]. CC80 was predominant in North African countries and CC88 was predominant in Sub-Saharan countries. CC5 was found to be endemic in African countries [31].

A high diversity of clonal lineages has been identified among methicillin-susceptible *S. aureus* (MSSA) strains isolated from animals (livestock, domestic, and wild animals) in Africa. CC1 and CC15 were frequently detected in African countries [32]. CC80-MRSA was isolated from nasal swabs of sheep in Côte d’Ivoire, Algeria, and Tunisia [33,34,35]. *S. aureus* belonging to CC88-MRSA, CC5-MRSA and CC22-MRSA were also found in bovine mastitis in Egypt [36].

In Algeria, studies describing the clonal diversity of *S. aureus* in both, humans and animals, were limited or focused on MRSA in some regions or restricted to specific animal species. It has been reported that the clone ST80-MRSA was predominant in human isolates [15,25,37]. In these studies, isolates carried SCC*mec* elements of type IV, II, or V and PVL genes have been found. In livestock, few studies reported that PVL-positive ST80-MRSA-IV and ST152-MSSA were recognized in camels and sheep [35]. CC130-MSSA was mostly found in sheep and goat mastitis [38,39]. There are only a few publications about the clonal diversity of *S. aureus* isolated from food matrices in Algeria [40].

The aim of the present study was to investigate the clonal diversity and pathogenicity of *S. aureus* isolates and highlight their origins, lineages, and possible dissemination alongside the food chain. In this study, clonal lineages of MSSA and MRSA isolates collected from human and food samples in northern Algeria were characterized using DNA microarrays, and provided useful data on their virulence factor genes and antibiotic resistance-associated genes.

## 2. Materials and Methods

### 2.1. Sample Collection

The sampling procedure of this study was performed between January 2017 to January 2018 in two provinces (“wilayas”) of Médéa and Ain-Defla in northern Algeria. The provinces of Médéa and Ain Defla are two neighboring regions located in the central region of Algeria at nearly 100 km from the capital Algiers covering an area of 8866 km² and 4897 km², respectively. The two regions are characterized by a temperate Mediterranean climate. The region of Ain Defla and the north of Médéa are characterized by cereal cultivation and bovine farming. The south of Médéa belongs to an agro-pastoral region with animal breeding with a focus on sheep farming.

In this study, 71 volunteers (veterinarians and farmers) having contact with livestock animals and others with less exposure to animals such as administrative clerks were screened for staphylococcal nasal carriage. The nasal samples were obtained from both nares with sterile cotton swabs and were transported to the laboratory for microbiological examination.

Furthermore, 112 food samples (raw milk, beef meat, sausages, chicken meat, and creamery cake) were collected from several commercial vendors. The samples were cooled and transferred to the laboratory for microbiological examinations. Thirty-six *S. aureus* isolated from clinical samples and nasal swabs were kindly provided from local health-care facilities and private labs in the two provinces and were collected during the same time of food sampling.

### 2.2. Ethical Statement

The study protocol was approved by the Medical Ethics Research Committee of the Yahia Farès University, Urban Pole, Médéa, Algeria, and from the managers of the hospital in which the study was conducted. Informed written consent was obtained from each participant in the study. Confidentiality and personal privacy were respected at all levels of the study. Collected data will not be used for any other purpose.

### 2.3. Isolation of Staphylococcus spp.

The bacteriological analyses of samples from humans (nasal swabs, clinical samples) were performed using conventional methods, as described previously [41,42].

Nasal swabs and clinical samples were inoculated into Brain Heart Infusion (BHI) broth (Oxoid Ltd., Basingstoke, UK) and incubated overnight at 37 °C. Initial broth culture was streaked on Mannitol Salt Agar (MSA) (Oxoid Ltd., Basingstoke, UK) and Columbia agar supplemented with 5% sheep blood and incubated at 37 °C for 24 to 48 h. The plates were inspected for characteristic morphology of staphylococci and suspicious colonies were identified using conventional methods (Gram staining, catalase, and coagulase reactions) in accordance with standard microbiological methods [43]. The food samples were processed according to EN ISO 6888 1-2 1999 [44] using Baird–Parker medium supplemented with 5% of egg yolk emulsion (Oxoid Ltd., Basingstoke, UK) and 0.5% potassium tellurite. Colonies with typical shape were selected for presumptive conventional phenotypic and biochemical identification using catalase and coagulase tests.

Colonies were conserved on BHI broth with 50% of glycerol as well as by freeze-drying method in skimmed milk until further investigations.

### 2.4. MALDI-TOF MS Identification

Matrix-assisted laser desorption-ionization time-of-flight mass spectrometry (MALDI-TOF MS) was used for species identification; the procedure was performed as described by Bizzini and Greub, 2010 [45].

Briefly, all suspected isolates were subcultured on Mueller–Hinton agar (Oxoid Ltd., Basingstoke, UK) supplemented with 5% sheep blood at 37 °C for 24 h. Pure colonies were put in 300 μL of water, vortexed and then precipitated with 900 µL ethanol (96% vol/vol) (Carl Roth GmbH, Karlsruhe, Germany). After centrifugation for 5 min at 10,000× *g*, the pellet was resuspended with a volume of 50 μL of 70% (vol/vol) formic acid (Sigma Aldrich Chemie GmbH, Steinheim, Germany) and 50 μL of acetonitrile (Carl Roth GmbH, Karlsruhe, Germany). After centrifugation for 5 min at 10,000× *g*, 1.5 μL of the supernatant was spotted onto a MTP 384 Target Plate Polished Steel TF (Bruker Daltonik GmbH, Bremen, Germany) and allowed to air dry at room temperature. A droplet of 2 μL of matrix solution (α-cyano-4-hydroxycinnamic acid) (Sigma-Aldrich Chemie GmbH, Taufkirchen, Germany) was added on top of each sample spot and again the spots were completely dried. Spectra recording was performed using an Ultraflex instrument (Bruker Daltonik GmbH, Bremen, Germany) and data were automatically analyzed using the Biotyper 3.1 software (Bruker Daltonik, Bremen, Germany).

### 2.5. Molecular Identification of Staphylococcus spp.

DNA was extracted from suspected *Staphylococcus* colonies using the HighPure PCR Template Preparation Kit (Roche Diagnostics Deutschland GmbH, Mannheim, Germany) according to the manufacturer’s instructions. DNA quantity and purity were determined using a NanoDrop™ 1000 spectrophotometer (Thermo Fisher Scientific, Wilmington, MA, USA).

A multiplex real-time PCR assay was performed to detect 16S rRNA, *nuc* and *mec*A genes corresponding to *Staphylococcus* spp., *S. aureus,* and MRSA, respectively [46]. The multiplex real-time PCR TaqMan assay was carried out with the LightCycler 480 Probes Master kits (Roche Diagnostic Deutschland GmbH, Mannheim, Germany) using the CFX-96 real-time PCR system (Bio-Rad, Hercules, CA, USA), which was used for thermocycling and fluorescence detection as described previously [46]. The real-time PCR amplification was performed in a total volume of 25 μL containing 10 μL of PCR master-mix (1×), 6.75 μL of primers (0.2 µM) and TaqMan probe (0.1 µM) mixture and 5 μL of template DNA (100 pg/µL); distilled water was added for a final volume of 25 μL.

### 2.6. DNA Microarray Analysis

The microarray carried 334 oligonucleotide probes for virulence-associated genes including genes encoding exotoxins and enterotoxins, immune evasion factors, microbial surface components recognizing adhesive matrix molecules (MSCRAMM) as well as resistance determinants and other target genes for typing markers such as species, SCC*mec*, capsule, and *agr* group typing markers. DNA microarray analysis was performed on the ArrayStrip platform using the *S. aureus* specific DNA microarray assay (StaphyType; Abbott (Alere Technologies GmbH), Jena, Germany) following the manufacturer’s instructions. Briefly, bacterial colonies were picked from an overnight culture on blood agar. The genomic DNA was extracted after bacterial cells lysis using the EZ1 system (Qiagen, Hilden, Düsseldorf, Germany). Purified nucleic acids were subjected to a linear primer elongation. Biotin-16-dUTP was integrated into the resulting amplicons. Labeled genomic DNA was hybridized to the probes of the microarray. After washing, horseradish-peroxidase-streptavidin conjugate was added followed by precipitation with a special dye. Then, a scanned image was obtained by a microarray reader and subsequently the data were analyzed automatically using software provided by Alere Technologies GmbH. According to difference in the intensity of the colors for each spot, results interpretation was done as “positive” (present), “negative” (absent) or “ambiguous” as described previously [10,47,48].

The clonal complex recognition was based on analysis of hybridisation profiles with regard to non-motile genes, as previously reported [49].

## 3. Results

In this study, 60 *S. aureus* were isolated and identified using MALDI-TOF MS and real-time PCR. Twenty-four strains were isolated from 112 food samples, 16 were isolated from 71 nasal swabs and 20 clinical strains were kindly provided from hospital and private labs collection. The origin and distribution of *S. aureus* are shown in Table 1.

Twenty-four *S. aureus* isolated from food samples were predominantly isolated from raw milk (10 isolates), minced beef meat (10 isolates), creamy cake (2 isolates), chicken meat (one isolate) and sausages (one isolate) (Table 1).

**Figure 1:**
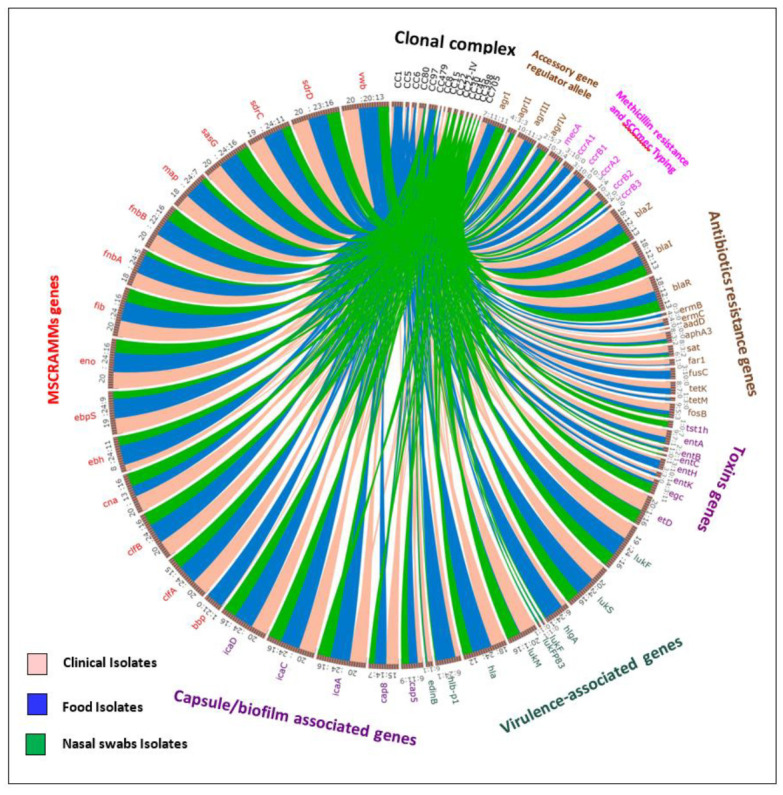
Graphic visualization of source of the isolates (clinical, food and nasal swabs) in circular layout [50], revealing the association between the MSCRAMMs genes, methicillin resistance and SCC*mec* Typing, capsule/biofilm-associated genes, virulence-associated genes, toxin genes, antibiotic resistance genes, accessory gene regulator allele, and the clonal complexes detected by microarray using interconnections with a variety of colored ribbons. The circumference (track) of the circle is divided into arcs of varying lengths according to the abundance of every single factor.

*Staphylococcus aureus* isolated in this study were grouped into 14 different clonal complexes using DNA microarray analysis (Table 1; Figure 1 and Table S1). Out of 24 *S. aureus* isolated from food, 6 different CCs were identified, while 9 different CCs were determined for 20 clinical isolates and those of nasal swabs (Table 1).

Seventeen (28.3%) out of 60 *S. aureus* isolates were identified as MRSA and they were mainly found in clinical isolates (10/17 (58.8%)). Only a minority was detected in food samples (3/17 (17.6%) and nasal samples (4/17 (23.5%)).

Regardless of their origin, 13 (21.7%) and 10 (16.7%) out of 60 *S. aureus* isolates belonged to CC1-MSSA and CC97-MSSA, respectively. These two CC were the most common clonal complexes.

The clinical isolates mainly belonged to CC80-MRSA (6/20 (30%)), followed by the CC1-MSSA and CC6-MRSA (3 /20 (15%) for each).

Ten (41.7%) and eight (33.3%) of 24 *S. aureus* isolated from food clustered mainly within CC1-MSSA and CC97-MSSA, respectively.

The occurrence of virulence-associated factors as detected by microarray analysis is summarized in Table 2 and Table 3.

Out of 60 *S. aureus*, 27 (45%) possessed the accessory gene regulator allele *agr* group III with a predominance of clinical isolates (19/27; 70.4%). Isolates having *agr*-III alleles belonged to CC1-MSSA, CC80-MRSA, CC30-MSSA, and CC5-MSSA (Table 2). The *agr*-I was detected in CC6-MRSA, CC22-MSSA, CC22-MRSA, CC45-MSSA, CC97-MSSA and CC398-MSSA. The *agr* group II alleles were found in isolates assigned to CC15-MSSA, CC479- MSSA and CC705-MSSA.

Panton-Valentine leukocidin genes *luk*F/*luk*S-PV were detected in 8 (13.3%) CC80 MRSA, which mainly originated from pus samples (4 isolates), urine and catheter (one isolate for each) of human origin. The genes *luk*F-PV (P83) and *luk*M were present in two isolates belonging to CC479-MSSA and CC705-MSSA. However, *luk*F*, luk*S*, hlg*A and *hla* encoding for hemolysins and leukocidin components were detected in all *S. aureus* isolates. The capsule type 5 gene cluster *cap*5 was carried by 24 (40%) isolates mainly belonging to CC5-MSSA, CC22-MSSA, CC22-MRSA and CC97-MSSA. Contrary, the *cap*8 gene cluster was harbored by 36 (60%) isolates mainly belonging to CC1-MSSA (13 isolates) and CC80-MRSA (8 isolates).

The *ica* operon ACD responsible for intercellular adhesion protein AB and biofilm PIA synthesis protein D was detected in all isolates, whereas the *bap* gene encoding surface protein involved in biofilm formation was found only in one CC15-MSSA isolate recovered from a pus sample of a patient with a hospital-acquired infection.

The gene encoding for toxic shock syndrome toxin 1 (*tst*1) was found in 8 human isolates assigned to CC22-MSSA and CC22-MRSA. Genes *et*A/*B* encoding for exfoliative toxins were not detected, while the *et*D gene was present in all CC80-MRSA isolates. The *edin*B gene encoding for epidermal cell differentiation inhibitor B was likewise detected in all isolates belonging to CC80-MRSA whereas, *edin*A and *edin*C genes were not detected.

Genes encoding for enterotoxins were detected in several isolates: The *sea* gene was found in 17 isolates (10 human isolates and 7 food isolates) belonging to CC1-MSSA, CC5-MSSA, CC6-MRSA, CC8-MSSA and CC30-MSSA. The *seb* and *sec* genes were identified in 5 and 2 isolates, respectively. The enterotoxin gene cluster *egc* was present in 17 isolates. The clinical isolates carrying the *sea* gene were predominantly collected from samples of community infections and the food isolates harboring this gene originated from milk and minced beef meat.

In all isolates we detected adhesion factors and genes encoding MSCRAMM [bone binding protein (*bbp*), clumping factors (*clf*A*/*B), collagen-binding adhesion (*cna*), cell wall-associated fibronectin-binding protein (*ebh*), cell surface elastin-binding protein (*ebp*S), enolase (*eno*), fibrinogen-binding protein (*fib*), fibronectin-binding protein A (*fnb*A), major histocompatibility complex class II (extracellular adherence protein), extracellular adherence protein (*map*), *S. aureus* surface protein G (*sas*G) and fibrinogen-/bone sialoprotein-binding protein C/D (*sdr*C/D) and van Willebrand factor-binding protein (*vwb*)] were detected in all isolates.

The genes responsible for antimicrobial resistance detected by microarray analysis are shown in Table 4.

The antibiotic resistance gene carriage was observed in different isolates regardless their origin.

Seventeen (28.3%) *S. aureus* isolates carried the *mec*A gene localized on SCC*mec* type IV elements. These isolates were identified as methicillin-resistant. The remaining *S. aureus* isolates (43/60; 71.7%) were methicillin-susceptible. The 17 MRSA isolates belonged to three different CCs (CC6, CC80, and CC22) and mainly originated from clinical samples (7 community-acquired MRSA and 3 hospital-acquired MRSA), 3 MRSA isolates from food, and 4 MRSA isolates came from nasal swabs.

Genes encoding for antibiotic resistance to β-lactamase (*bla*Z/I/R) were found in 71.7% (43/60) of isolates.

The *erm*(C) gene encoding for macrolide/clindamycin resistance was found in seven MRSA isolates, while only two MSSA isolates (food samples) assigned to CC97 carried the *erm*(B) gene.

Fusidic acid resistance gene *fus*C was detected in all CC1 isolates. The *tet*K gene associated with tetracycline resistance was identified in 15 *S. aureus* isolates (7 food and 8 clinical isolates). In one food isolate belonging to CC1-MSSA, *tet*K and *tet*M were detected simultaneously.

Additionally, the *aad*D gene, which is responsible for tobramycin resistance, was detected in one clinical isolate.

In addition to *mec*A gene carriage*,* clinical isolates that belonged to CC80-MRSA harbored antimicrobial resistance genes *bla*Z, *erm*C, *aph*A3, *sat*, *far*1 and *tet*K. They were recovered from pus samples (4 isolates), urine (1 isolate) and a catheter (1 isolate). CC97-MSSA isolated from food samples carried several antibiotic resistance genes (*bla*Z, *erm*B, *aph*A3, *sat*, *tet*M, and *tet*K genes). These strains were mainly isolated from milk samples (*n* = 5), cream cake (*n* = 2) and minced beef meat (*n* = 1) (Table 1).

## 4. Discussion

To estimate the potential health hazards caused by *S. aureus* transmission, it is imperative to study their origins, lineages and dissemination simultaneously on humans, in the environment, on animal farms and alongside the food chain.

The present study was aimed to provide relevant information (clonal complex lineages, *agr* types, virulence and antimicrobial resistance-associated genes) about *S. aureus* isolated from a variety of human clinical, nasal and food samples in northern Algeria based on DNA microarray assay. This study focused only on two provinces (Médéa and Ain-Defla) in Algeria and hence our results cannot be generalized for a larger scale.

In this study, 24 *S. aureus* from food sources originated predominantly from raw milk and minced beef meat and only three of them were identified as MRSA. These findings corroborated several studies reporting low percentages of MRSA in food samples [51,52,53].

The majority of clinical isolates of *S. aureus* were recovered from pus and suppurative infections in which 50% were identified as MRSA (7 CA-MRSA and 3 HA-MRSA). It is known that *S. aureus* isolates are considered as a principal pathogenic agent in skin and soft tissue infections [54].

Out of 16 *S. aureus* isolated from nasal carriage from individuals with contact to livestock animals, four (25%) were identified as MRSA. These findings were lower compared to previous reports conducted in Algeria on resembled populations [35,55].

Molecular genotyping of isolated *S. aureus* resulted in assignment to a high variety of CCs. The general trend showed that CCs in clinical or food samples were closely similar, in contrast to those detected in nasal carriage. The lineages found in food isolates mostly belonged to CC1-MSSA and CC97-MSSA. The majority of CC97 (62.5%) originated from milk samples. Previous studies indicated that *S. aureus* isolated from bovine milk belonged to CC97 and were mainly represented by isolates from ruminants [9,10,20,56,57]. CC97 strains are occasionally isolated from humans [58] and rarely found as MRSA [59].

Two human isolates belonged to the common bovine lineage CC97, suggesting a possible jump from cattle to humans, as reported previously [60]. A previous work carried out in the same region as the present study indicated that CC97 is associated with bovine subclinical mastitis [55], suggesting a possible origin of these milk samples from infected udders without rigorous surveillance.

The majority of CC1-MSSA isolates originated from meat samples and the remaining came from milk samples which in agreement with a previous study conducted by Wu et al., 2018 reported that most of *S. aureus* isolated from retail meat and meat products belonged to CC1-MSSA [61]. The CC1-MSSA is thought to be of human origin and began to emerge within bovine *S. aureus* populations causing mastitis [62], which could subsequently contaminate milk, as previously reported [9].

In this study, three MRSA belonging to CC6-MRSA-IV and CC80-MRSA-IV were isolated from food samples (minced beef meat). These lineages are likely to be found in human samples [25,63] so that this observation might indicate a possible contamination by humans at the slaughterhouse or during processing [64]. Another study showed that commercially distributed meat could play a role in the spreading of CA-MRSA [65]. However, there is no evidence that the transmission of such lineages (lacking enterotoxin genes) would increase the risk of foodborne infections [66].

Six MRSA strains isolated from clinical samples belonged to CC80-MRSA-IV. They were PVL positive and carried SCC*mec* type IV. This lineage has previously been found in clinical samples in Algeria [15,25,37,67], Tunisia [68], Egypt [16], Europe [23], North America [18], the Middle East [69] and Australia [19]. The results suggest that this clonal complex is a highly prevalent MRSA lineage that has already spread across the North African countries, Egypt and the Middle East and that also can be found sporadically beyond that region.

Seven of the clinical MSSA strains were assigned to CC1, CC5, and CC15 which in agreement with previously reported diversity of CCs in MSSA isolated from patients with infected diabetic foot ulcer in which MSSA isolates belonged to CC1 and CC15 [37].

*Staphylococcus aureus* from nasal swabs essentially belonging to CC22 (CC22-MSSA and CC22-MRSA); only one isolate belonged to CC80-MRSA IV. The results coincide with previous report, which found that MRSA in nasal carriers on farms belonged to CC22-MRSA-IV and CC80-MRSA-IV [55]. The prominent epidemic CC22 lineage is known as a typical widespread human-associated clone, and there are several distinct MRSA strains that are responsible for both, hospital-acquired and community-acquired MRSA infections [59,70].

One nasal carriage isolate was assigned to CC398-MSSA. The results of a former study strongly suggested that LA-MRSA CC398 originated in humans as MSSA [71]. This clone has been recovered also from food of animal origin [72]. *Staphylococcus aureus* of ST398 is known to have comparable low host specificity and is able to colonize or cause infections in a broad variety of animal species, including humans [59,73]. It is worth to note that isolated CC398-MSSA lacked many virulence factors such as the Panton–Valentine leukocidin toxin or enterotoxin genes; also, no antibiotic resistance determinants were detected. In fact, the transmission of some clones from animals to humans and *vice versa* is accompanied by the acquisition of adaptation factors and at the same time loss of certain virulence factors [73,74].

The present findings on human nasal carriage revealed that one *S. aureus* belonged to CC705-MSSA. It was reported that this clone was not of human origin but it is a common lineage in cattle [60,75]. In the same way, Agabou et al., 2017 found only two CC705-MSSA isolates in Algerian cattle nasal carriage without any detection of this clone in noses of farm workers [35]. Furthermore, it was reported that CC705 (ST705) has emerged as a dominant lineage derived from bovine milk worldwide [56]. Thus, we assume a transmission from an animal or food source to the human carrier.

Two isolates originating from nasal carriage were assigned to CC15-MSSA. Monecke et al. (2009) reported that CC15-MSSA are mainly isolated from healthy carriers [76]. In addition, it was suggested that MRSA from this lineage are extremely rare [59]. Two other human isolates assigned to CC30-MSSA were identified in this study. A previous study showed that CC30 is an important clonal complex from which virulent MSSA as well as HA and CA-MRSA originated [59].

In the present study, other *S. aureus* lineages, such as CC30 and CC45, were recovered from nasal carriers. These CCs were described in the past among other clusters of strains colonizing healthy individuals in Europe [73,76,77].

In this study, Panton–Valentine leukocidin genes *luk*F*/luk*S*-*PV were detected in 8 *S. aureus* isolated from clinical samples and belonging to CC80-MRSA. In concordance, a study described that all MRSA harboring *luk*F*/luk*S*-*PV belonged to CC80 [37]. Other reports confirming that PVL gene carriage associated with CC80 is widely distributed in North African countries, including Algeria [15,25].

From the three MRSA isolated from food samples, we found only one PVL-positive. Recently, Mairi et al., 2019 found also some MRSA-PVL positive isolates from food belonging to the ST80-IV CA-MRSA clone [52]. However, MRSA PVL-positive clonal lineages are rare in milk products [78] or in animals [35,79].

The leukocidin genes *luk*F-PV(P83) (F component from hypothetical leukocidin of ruminants) and *luk*M were detected in CC705 (nasal carriage isolate) and CC479 (food isolate). Schlotter et al., 2012, found that the majority of CC479 *S. aureus* isolates from milk of dairy herds harbored the leukocidin genes *luk*F-(P83)/*luk*M [80]. In a comparative study of 456 strains of *S. aureus* isolated from milk of bovine intramammary infections and bulk tanks obtained from 12 European countries, it was reported that isolates were predominantly assigned to CC705 [81]. In addition, several studies reported that CC705 is a common bovine clone among bovine mastitis *S. aureus* isolates [55,60,82] and from nasal carriage in cattle [35]. The detection of leukocidin genes *luk*F-PV (P83)/*luk*M in a human nasal swab isolate CC705 suggests zoonotic transmission from bovine to humans in close contacts.

In this study, *sea* and *seh* genes were notably detected in CC1 MSSA isolated from food samples. Staphylococcal food poisoning (SFP) is an intoxication caused by ingestion of contaminated foods (milk, cream filled pastries, sandwich filling, sausages, ground meat, salads, and cooked meals) containing sufficient quantity of staphylococcal enterotoxins (SEs). The most important enterotoxins involved in SFP are the classical SEA, and the SEH [3]. Monecke et al., 2011 found that all CC1-MRSA isolates harbored the enterotoxin gene *seh* [59]. Others found the *seh* gene in *S. aureus* isolates from milk and milk products [78]. Also, Jørgensen et al., 2005 reported that SEH was responsible for milk-associated food-poisoning outbreaks [83].

It has been reported that isolates harboring the gene encoding for toxic shock syndrome toxin 1 (*tst*1) in combination with other toxin genes were highly pathogenic for humans [84]. In this study, isolates possessing the *tst*1 gene were negative for *luk*FS-PV*, luk*F-PV *(P83), luk-*M*, sea, seb* and *sec* genes.

Biofilm formation is regulated by expression of polysaccharide intracellular adhesion (PIA) protein, which mediates cell-to-cell adhesion and is the product of the *ica* locus containing *ica*A/B/C/D [85]. The operon *ica*ACD was detected in all isolates. The *bap* gene encoding surface protein involved in biofilm formation was found only in one CC15-MSSA isolate. All MRSA SCC*mec* type IV isolates possessed the *ica*ACD genes responsible for biofilm formation and carried the MSCRAMMs genes. In concordance, Mirani et al., 2013 found that 98.3% of foodborne MRSA isolates carried the *ica*A/SCC*mec* IV profile [86], while an association of SCC*mec* type V and *ica*A gene was also reported [87,88].

The present study showed a high rate of MRSA in clinical samples (50%). The national rate in Algeria was approximately 35% for hospitalized patients (www.sante.dz/aarn/documents/pdf/rapport16.pdf, accessed on 27 September 2021). Several Algerian studies focusing on human isolates showed that the MRSA rate has increased almost tenfold in just two decades from less than 5% in 1996–1997 [89] to 50% in 2011 [25] and 62.2% in 2014 [90].

In this study, all MRSA isolates carried the SCC*mec* IV type. Previous Algerian studies reported that MRSA strains mostly harbored the SCC*mec* IV type [15,25,67,91]. Additionally, others SCC*mec* types were found at lower rates: 43.75% of MRSA carried SCC*mec* V type [91]. SCC*mec* II in one isolate [15], SCC*mec* type III [25,67], while Alioua et al. (2014) found more SCC*mec* type III compared to SCC*mec* type IV [90].

Resistance to antibiotics can result from the imprudent use of antimicrobial agents in human and animals. In this study, 71.7% of all *S. aureus* and 88.2% of MRSA isolates carried the penicillinase operon *bla*Z. Previous Algerian studies reported penicillin resistance in 96 to 100% of clinical isolates [92] and in nasal human carriage, 91.6% of isolates carried the *bla*Z gene [35].

The detection of genes encoding resistance towards tetracycline (*tet*M and *tet*K) was not surprising, as this broad-spectrum antibiotic is widely used by Algerian veterinarians and farmers [93]. A resistance rate as high as 85% to tetracycline among MRSA strains isolated from poultry was recently reported [94]. In addition, tetracycline resistance could be expected in other bacterial genera as a consequence of the extensive use of tetracycline in livestock [93].

The putative/questionable fosfomycin resistance gene *fos*B was described by Djahmi et al., 2013, who found a high rate of clinical isolates assigned to several CCs (ST239-MRSA, ST80-MRSA, CC1-, CC15-, CC121-, CC9-, CC54- and ST152-MSSA) harboring *fos*B [37]. Indeed, it was reported to be specific for certain clonal complexes such as CC5, CC8, CC12, CC15, CC20, CC25, CC30 and *S. argenteus* ST1850 [49].

Monecke et al., 2011 found that nearly all CC80-MRSA-IV isolates carried the *aph*A3 and *sat* and plasmid harbored genes *bla*Z, *tet*K and *far*1. Additional resistance genes *erm*C were rarely detected [59]. In concordance, all CC80-MRSA-IV isolated in this study harbored the *aph*A3 and *sat* genes. 87.5% carried the genes *far1, tet*K and *bla*Z and 25% carried the *erm*C gene. Others reports showed also that MRSA-ST80 isolates can be resistant to fusidic acid, tetracycline and kanamycin [95,96]. Ultimately, we considered CC80-MRSA-IV isolates of the present study as multidrug-resistant.

## 5. Conclusions

The DNA microarray results obtained in this study showed that CC 80-MRSA- IV is a significant pathogen among clinical isolates. This clone was found to be associated with many virulence factors (*luk*F*/luk*S-PV, *edin*B and *sea*) and antibiotics resistance genes (*sat, aph*A3*, far*1*, tet*K and *bla*Z). In addition, food isolates mostly assigned to CC1 and CC97 were found to harbor enterotoxins genes and antibiotic resistance genes. It was also observed that almost all clones were found in their typical host, but interestingly, some nasal carriers had isolates assigned to CC705 thought to be absent in humans. Our data suggest a possible jump from animals to humans and constitute a dissemination risk into the community. *Staphylococcus aureus* continues to be a serious human health challenge and the detection of MRSA strains among food isolates should be considered as a potential public health risk. It is necessary to monitor the health status of animals and improve hygiene conditions in the food chain to limit the dissemination of pathogenic and multidrug-resistant strains. Therefore, it is crucial to revise antibiotic prescriptions for rational use in human and animal infections.

## Figures and Tables

**Table 1:** Assignment of 60 *S. aureus* isolated from different samples and their origin.

Clonal Complex	Source of Isolates								Total CC
	Clinical Samples			Food Samples			Nasal Swabs		
	Source	Isolates Number	Total	Source	Isolates Number	Total	Isolates Number	Total	
CC1-MSSA	Blood culture	1	3	Raw milk	4	10	-	-	13
	Pus	1		Chicken meat	1				
	Surgical wound	1		Minced beef meat	4				
				Sausages	1				
CC5-MSSA	Urine	1	2	Minced beef meat	2	2	-	-	4
	Vaginal discharge	1							
CC6-MRSA IV	Catheter	2	3	Minced beef meat	2	2	-	-	5
	Pus	1							
CC80-MRSA IV	Sperm	1	6	Minced beef meat	1	1	1	1	8
	Pus	4							
	Urine	1							
CC97-MSSA	Throat fluid	1	1	Creamy cake	2	8	1	1	10
				Raw milk	5				
				Minced beef meat	1				
CC15-MSSA	Pus	2	2	-	-	-	2	2	4
CC22-MRSA-IV	Pus	1	1	-	-	-	3	3	4
CC30-MSSA	Pus	1	1	-	-	-	1	1	2
CC8-MSSA	Urine	1	1	-	-	-	-	-	1
CC479-MSSA	-	-	-	Raw milk	1	1	-	-	1
CC22-MSSA	-	-	-	-	-	-	4	4	4
CC398-MSSA	-	-	-	-	-	-	1	1	1
CC45-MSSA	-	-	-	-	-	-	2	2	2
CC705-MSSA	-	-	-	-	-	-	1	1	1
Total			20			24		16	60

**Table 2:** Distribution of virulence-associated genes in *S. aureus* isolates.

Clonal Complex (*n*)	Origin * (*n*)	*agr* Group (*n*)	Virulence-Associated Genes									Capsule/Biofilm-Associated Genes			Toxin Genes							
			*luk*FS-PV	*luk*F-PV (P83)	*luk*-M	*luk*F-*hlg*	*luk*S-*hlg*	*hlg*A	*hla*	*hlb*	*edin*B	*cap*5	*cap*8	*ica*ACD	*sea*	*seb*	*sec*	*seh*	*sek*	*egc*	*tst1*	*etD*
CC1-MSSA (13)	C (3)	*agr* III (3)	-	-	-	3	3	3	3	3	-	-	3	3	3	1	-	3	3	-	-	-
	F (10)	*agr* III (10)	-	-	-	10	10	10	10	10	-	-	10	10	3	-	-	10	3	-	-	-
CC5-MSSA (4)	C (2)	*agr* III (2)	-	-	-	2	2	2	2	2	-	2	-	2	2	-	-	-	-	2	-	-
	F (2)	*agr* III (2)	-	-	-	2	2	2	2	2	-	2	-	2	2	-	-	-	-	2	-	-
CC6-MRSA IV	C (3)	*agr* I (3)	-	-	-	3	3	3	3	3	-	-	3	3	3	-	-	-	-	-	-	-
	F (2)	*agr* I (2)	-	-	-	2	2	2	2	2	-	-	2	2	2	-	-	-	-	-	-	-
CC8-MSSA (1)	C (1)	*agr* I (1)	-	-	-	1	1	1	1	1	-	1	-	1	1	-	1	-	-	-	-	-
CC15-MSSA (4)	C (2)	*agr* II (2)	-	-	-	2	2	2	2	-	-	-	2	2	-	-	-	-	-	-	-	-
	N (2)	*agr* II (2)	-	-	-	2	2	2	2	-	-	-	2	2	-	-	-	-	-	-	-	-
CC22-MSSA (4)	N (4)	*agr* I (4)	-	-	-	4	4	4	4	4	-	4	-	4	-	-	-	-	-	4	4 ^§^	-
CC22-MRSA- IV (4)	C (1)	*agr* I (1)	-	-	-	1	1	1	1	1	-	1	-	1	-	-	-	-	-	1	1 ^§^	-
	N (3)	*agr* I (3)	-	-	-	3	2	3	2	3	-	3	-	3	-	-	-	-	-	3	3 ^§^	-
CC30-MSSA(2)	C (1)	*agr* III (1)	-		-	1	1	1	1	-	-	-	1	1	-	-	-	-	-	1	-	-
	N(1)	*agr* III (1)	-	-	-	1	1	1	1	1	-	-	1	1	1	-	-	-	-	1	-	-
CC45-MSSA (2)	N(2)	*agr* I (2)	-	-	-	2	2	2	2	-	-	-	2	2	-	-	1	-	-	2	-	-
CC80-MRSA- IV (8)	C (6)	*agr* III (6)	6	-	-	6	6	6	6	6	6	-	6	6	-	-	-	-	-	-	-	6
	N (1)	*agr* III (1)	1	-	-	1	1	1	1	1	1	-	1	1	-	-	-	-	-	-	-	1
	F (1)	*agr* III (1)	1	-	-	1	1	1	1	1	1	-	1	1	-	-	-	-	-	-	-	1
CC97-MSSA (10)	C (1)	*agr* I (1)	-	-	-	1	1	1	1	1	-	1	-	1	-	1	-	-	-	-	-	-
	N (1)	*agr* I (1)	-	-	-	1	1	1	1	1	-	1	-	1	-	1	-	-	-	-	-	-
	F (8)	*agr* I (8)	-	-	-	8	8	8	8	8	-	8	-	8	-	2	-	-	-	-	-	-
CC398-MSSA (1)	N (1)	*agr* I (1)	-	-	-	1	1	1	1	1	-	1	-	1	-	-	-	-	-	-	-	-
CC479- MSSA	F (1)	*agr* II (1)	-	1	1	1	1	1	1	1	-	-	1	1	-	-	-	-	-	1	-	1
CC705-MSSA	N (1)	*agr* II (1)	-	1	1	1	1	1	1	1	-	-	1	1	-	-	-	-	-	1	-	-

* C: clinical isolates; F: food isolates; N: isolates from nasal swabs. ^§^ toxic shock syndrome toxin-1 *tst*1 (“human” allele).

**Table 3:** Distribution of MSCRAMM genes in *S. aureus* isolates.

Clonal Complex (*n*)	Origin * (*n*)	*agr* Group (*n*)	MSCRAMM Genes													
			*bbp*	*clf*A/B	*cna*	*ebh*	*epbs*	*Eno*	*fib*	*fnb*A	*fnb*B	*map*	*sas*G	*sdr*C	*sdr*D	*vwb*
CC1-MSSA (13)	C (3)	*agr* III (3)	3	3	3	3	3	3	3	3	3	3	3	3	3	3
	F (10)	*agr* III (10)	10	10	10	10	10	10	10	10	9	10	10	10	10	10
CC5-MSSA (4)	C (2)	*agr* III (2)	2	2	-	2	2	2	2	2	2	2	2	2	2	2
	F (2)	*agr* III (2)	2	2	-	2	2	2	2	2	2	2	2	2	2	2
CC6-MRSA IV (5)	C (3)	*agr* I (3)	3	3	3	3	3	3	3	3	3	3	3	3	3	3
	F (2)	*agr* I (2)	2	2	2	2	2	2	2	2	2	2	2	2	2	2
CC8-MSSA (1)	C (1)	*agr* I (1)	1	1	-	1	1	1	1	1	1	1	1	1	1	1
CC15-MSSA (4)	C (2)	*agr* II (2)	2	2	-	2	2	2	2	2	2	2	2	2	2	2
	N (2)	*agr* II (2)	2	2	-	2	2	2	2	2	2	2	2	2	2	2
CC22-MSSA (4)	N (4)	*agr* I (4)	4	4	4	4	4	4	-	4	-	4	4	4	4	4
CC22-MRSA- IV (4)	C (1)	*agr* I (1)	1	1	1	-	1	1	-	1	-	1	1	1	1	1
	N (3)	*agr* I (3)	3	3	3	-	3	3	-	3	-	3	3	3	3	3
CC30-MSSA (2)	C (1)	*agr* III (1)	1	1	1	1	1	1	-	1	-	1	-	1	1	1
	N(1)	*agr* III (1)	1	1	1	1	1	1	-	1	-	1	-	1	1	1
CC45-MSSA (2)	N(2)	*agr* I (2)	2	2	2	2	2	2	-	2	2	2	-	2	-	2
CC80-MRSA- IV (8)	C (6)	*agr* III (6)	6	6	-	6	6	6	6	6	6	6	6	6	6	6
	N (1)	*agr* III (1)	1	1	-	1	1	1	1	1	1	1	1	1	1	1
	F (1)	*agr* III (1)	1	1	-	1	1	1	1	1	1	1	1	1	1	1
CC97-MSSA (10)	C (1)	*agr* I (1)	1	1	-	1	1	1	1	1	1	1	1	1	1	1
	N (1)	*agr* I (1)	1	1	-	1	1	1	1	1	1	1	1	1	1	1
	F (8)	*agr* I (8)	6	8	-	8	8	8	8	8	7	8	8	8	8	8
CC398-MSSA (1)	N (1)	*agr* I (1)	-	1	1	1	1	1	-	1	1	1	-	1	1	1
CC479- MSSA (1)	F (1)	*agr* II (1)	-	1	1	1	1	1	1	1	1	1	1	1	-	1
CC705-MSSA (1)	N (1)	*agr* II (1)	1	1	-	1	1	1	1	1	-	1	-	1	-	1

* C: clinical isolates; F: food isolates; N: isolates from nasal swabs.

**Table 4:** Antimicrobial resistance-associated genes detected in 60 *S. aureus* isolates.

Clonal Complex (*n*) *	Origin (*n*) *	Antimicrobial Resistance Genes (*n*)												
		*mec*A	SCC*mec*	*bla*Z	*erm*B	*erm*C	*aph*A3	*aad*D	*sat*	*fus*C	*far*1	*tet*M	*tet*K	*fos*B
CC1-MSSA (13)	F (10)	-	*ccr*AB1 (10)	3	-	-	1	-	1	10	-	-	-	-
	C (3)	-	*ccr*AB1 (3)	2	-	-	-	-	-	3	-	1	1	-
CC5-MSSA (4)	C (2)	-	*-*	-	-	-	-	-	-	-	-	-	-	2
	F (2)	-	*-*	-	-	-	-	-	-	-	-	-	-	2
CC6-MRSA IV (5)	C (3)	3	*ccr*AB2 (3)	3	-	3	-	-	-	-	-	-	-	3
	F (2)	2	*ccr*AB2 (2)	1	-	2	-	-	-	-	-	-	-	2
CC8-MSSA (1)	C (1)	-	-	1	-	-	-	-	-	-	-	1	-	1
CC15-MSSA (4)	C (2)	-	-	2	-	-	-	1	-	-	-	-	2	2
	N (2)	-	-	2	-	-	-	-	-	-	-	-	-	2
CC22-MSSA (4)	N (4)	-	-	4	-	-	-	-	-	-	-	-	-	-
CC22-MRSA- IV (4)	C (1)	1	*ccr*AB2 (1)	1	-	-	-	-	-	-	-	-	-	-
	N (3)	4	*ccr*AB2 (3)	3	-	-	-	-	-	-	-	-	-	-
CC30-MSSA (2)	C (1)	-	-	1	-	-	-	-	-	-	-	-	-	1
	N (1)	-	-	1	-	-	-	-	-	-	-	-	-	1
CC45-MSSA (2)	N (2)	-	-	2	-	-	-	-	-	-	-	-	-	-
CC80-MRSA- IV (8)	C (6)	6	*ccr*AB2 (6)	6	-	1	6	-	6	-	6	-	6	-
	N (1)	1	*ccr*AB2 (1)	-	-	-	1	-	1	-	-	-	-	-
	F (1)	1	*ccr*AB2 (1)	1	-	1	1	-	1	-	1	-	1	-
CC97-MSSA (10)	C (1)	-	-	1	-	-	1		1	-	-	-	-	-
	N (1)	-	-	1	-	-	1		1	-	-	-	-	-
	F (8)	-	-	8	2	-	2		2	-	-	1	4	-
CC398-MSSA (1)	N (1)	-	-	-	-	-	-	-	-	-	-	-	-	-
CC479-MSSA (1)	F (1)	-	-	-	-	-	-	-	-	-	-	-	1	-
CC705-MSSA (1)	N (1)	-	-	-	-	-	-	-	-	-	-	-	-	-

* N: number of isolates; C: Clinical samples; F: Food samples; N: Nasal samples.

## Data Availability

The data presented in this study are available on request from the corresponding author.

## Supplementary Materials

The following are available online at https://www.mdpi.com/article/10.3390/pathogens10101276/s1, Table S1: The results of Microarray assay analysis for 60 *S. aureus*.
